# The *Hsp70* Gene Family in *Solanum tuberosum*: Genome-Wide Identification, Phylogeny, and Expression Patterns

**DOI:** 10.1038/s41598-018-34878-7

**Published:** 2018-11-09

**Authors:** Jia Liu, Xin Pang, Yuan Cheng, Yuhe Yin, Qiang Zhang, Wenbin Su, Bing Hu, Qinwei Guo, Si Ha, Jianping Zhang, Hongjian Wan

**Affiliations:** 1 Wulanchabu Academy of Agricultural and Husbandry Sciences, Wulanchabu, 012000 Inner Mongolia China; 2 Plant Protection Institute, Inner Mongolia Academy of Agricultural and Animal Husbandry Sciences, Hohhot, Inner Mongolia 010031 China; 3 Suzhou Polytechnic Institute of Agriculture, Suzhou, 215008 Jiangsu China; 4 State Key Laboratory Breeding Base for Zhejiang Sustainable Pest and Disease Control, Institute of Vegetables, Zhejiang Academy of Agricultural Sciences, Hangzhou, China; 5 Quzhou Academy of Agricultural Sciences, Quzhou, 324000 Zhejiang China

## Abstract

Heat shock protein 70 (Hsp70) family members play important roles in protecting plants against abiotic stresses, including salt, drought, heat, and cold. In this study, 20 putative *StHsp70* genes were identified in potato (*Solanum tuberosum* L.) through the integration of the gene structures, chromosome locations, phylogenetic relationships, and expression profiles. These *StHsp70* genes were classified into five sub-families based on phylogenetic analysis. Chromosome mapping revealed that they were unevenly and unequally distributed on 10 of the 12 chromosomes. Furthermore, segmental and tandem duplication events contributed to the expansion of the *StHsp70* genes. Phylogenetic tree of the *HSP70* genes from potato and other plant species revealed multiple sub-families. These findings indicated a common ancestor which had generated diverse sub-families prior to a mono-dicot split. In addition, expression analysis using RNA-seq revealed that the majority of these genes were expressed in at least one of the tested tissue, and were induced by *Phytophthora infestans*. Then, based on qRT-PCR analysis, the results showed that the transcript levels of some of the *StHsp70* genes could be remarkably induced by such abiotic and hormone stresses, which indicated their potential roles in mediating the responses of potato plants to both abiotic and biotic stress conditions.

## Introduction

In recent decades, global warming has caused serious economic losses in crop yields worldwide. Heat stress is one of the most important abiotic factors which threaten the growth and development of plants^1^. Some research studies have revealed that heat shock proteins (*HSPs*) have the ability to increase the thermo-tolerances of plants in order to help them cope with heat shock^2^. These heat shock proteins can be divided into five major families based on their molecular masses. The small heat shock proteins are referred to as *sHSP*, *HSP60*, *HSP70*, *HSP90*, and *HSP100*^3^. Among these, the *HSP70* proteins are highly conserved and widespread^4,5^, and were first identified and characterized in the 1960s^6^. The *HSP70* genes consist of three parts as follows: The first is an approximate 44-kD N-terminal ATPase domain (NBD); the second is an approximate 18-kD substrate binding domain (SBD); and the third one is an approximate 10-kD variable C-terminal “lid”^7^.

The *HSP70* proteins have been ubiquitously discovered in bacteria, plants, and humans^8^. In plants, they play a crucial role in the responses to biotic stresses, such as pathogen and nematodes^9^, and abiotic stresses, such as environmental factors, heat, cold, drought, and salinity^10^. The *HSP70* proteins are distributed in different plant cell organelles, and participate in diverse cellular functions in order to maintain the homeostasis of the plant protein. The *HSP70* gene family in plants has been classified into four subfamilies based on the subcellular localization as follows: cytosol, endoplasmic reticulum (ER), plastids, and mitochondria^5^. Furthermore, *NtHSP70-1* has been localized in *Nicotiana tabacum*^10^. In addition, the amplification and diversification evolution of the *HSP70s* from lower plants to higher plants have been determined, and were observed to maintain the homeostasis of protein folding through co-chaperones and cooperation^11^.

At the present time, the biological functions of the *HSP70* genes in plant response to abiotic and biotic stresses have been characterized in many plant species. For example, in *Arabidopsis*, mutant plants (*cPHSC70-1* and *cPHSC70-2* knockout) showed defective phenotypes after the seeds were germinated under high temperature conditions^12^. Furthermore, previous studies have also found that *AtHSP70-15* deficient in Arabidopsis increased mortality after heat treatments, and the over-expression of *AtHSP70-1* increased the thermo-tolerance of plants undergoing heat stress^13^. In addition, previous research results have also revealed that the *BIP1* gene encoding *HSP70* has the ability to regulate immunity in rice by *Xa21*-mediation^14^. It was observed that the *BIP* genes were responsive to female gametophytes in *Arabidopsis thaliana*^15^, as well as water stress in tobacco plants^16^. Furthermore, *BIP1* was observed to take part in rice seed development^17^. Recently, some study results have revealed that a relationship between the *ABA* and *HSP70* induced antioxidant responses in maize^18^. In tobacco plants, *NtHSP70-1* has been induced using *ABA* treatments, which has been found to increase in stress tolerance and cytoprotection^19^. However, the biological functions of many HSP70 genes have not yet been identified in many plants including potato.

Potato (*Solanum tuberosum* L.) is one of most important crops, with large-scale production all over the world. Potato provides not only carbohydrates and dietary fiber (skin), but also several vitamins, polyphenols, and minerals for human consumption. Therefore, since potatoes provide essential nutrients, they are very helpful for the human production of antioxidant defense systems. Also, colored potatoes have more anthocyanin and β-carotene^20^. Currently, potato production is under the threat of biotic stress conditions and environmental factors. Recently, the sequencing of the potato genome was available^21^. Therefore, identification of the potential of the *HSP70* gene family in responses to abiotic stresses are now considered to be feasible in potato.

In the current study, a total of 20 *HSP70* genes were identified using a bioinformatic method. The expression profiles of the *HSP70* gene family in response to abiotic and hormonal stress conditions were revealed. These stresses included heat, cold, drought and salt stresses, as well as indole-3-acetic acid (IAA), abscisic acid (ABA), gibberellin A_3_ (GA_3_), and salicylic acid (SA) stresses. The results of this studies’ experimental testing will be potentially helpful in furthering the understanding of the function and evolutionary history of the HSP70s in potato.

## Results

### Identification of the *HSP70* gene family in potato

In this study, the entire potato genome was downloaded and used to construct a local database. The HMM profile of the *HSP70* domain (PF00012) from Pfam (http://www.sanger.ac.uk/Software/Pfam) was utilized to search the candidate potato *HSP70* proteins as a query. A total of 25 putative *HSP70* genes in potato were identified. Among the 25 putative *HSP70* genes, five candidate sequences were removed due to their redundancy or incomplete domains. Then, the remaining 20 *HSP70* genes (*StHsp70-1* to *StHsp70-20*) in potato were identified. The detailed information of the *StHsp70* genes is shown in Table 1, including the names, chromosome locations, intron numbers, protein lengths, molecular weights, and pI values of the genes. The predicted molecular weight was found to vary from 53.27 KDa to 98.96 KDa. The *StHsp70* genes’ encoded proteins ranged from 486 to 890 amino acids. The pI of the *StHsp70* genes were determined to range from 5.03 (*StHsp70-8*) to 5.95 (*StHsp70-4*).

**Table 1:** The characteristics of Hsp70 genes in potato.

Gene name	Sequence ID	Chromosome	ORF (bp)	Intron number	Length (aa)	MW (KDa)	pI
StHsp70-1	PGSC0003DMP400043144	1:80158.5-80162.9	2121	7	706	75.15	5.16
StHsp70-2	PGSC0003DMP400054653	12:50389.4-50395.4	2613	9	870	95.99	5.34
StHsp70-3	PGSC0003DMP400054661	12:50378.3-50384.1	2550	8	849	93.57	5.23
StHsp70-4	PGSC0003DMP400005638	1:82924.1-82928.6	2025	5	674	72.41	5.95
StHsp70-5	PGSC0003DMP400018878	1:82849.8-82853.6	2034	5	677	72.78	5.82
StHsp70-6	PGSC0003DMP400027887	7:42050.3-42058.2	2673	12	890	98.96	5.83
StHsp70-7	PGSC0003DMP400000877	11:38747.6-38749.9	1959	1	652	71.31	5.14
StHsp70-8	PGSC0003DMP400052917	6:56528.0-56531.0	1947	1	648	71.02	5.04
StHsp70-9	PGSC0003DMP400033372	10:51360.2-51363.5	1944	1	647	70.89	5.03
StHsp70-10	PGSC0003DMP400025002	3:57412.2-57414.4	1965	1	654	71.89	5.21
StHsp70-11	PGSC0003DMP400049888	12:49581.3-49583.7	1920	1	639	70.80	5.42
StHsp70-12	PGSC0003DMP400015694	9:1958.6-1962.0	1950	1	649	71.24	5.13
StHsp70-13	PGSC0003DMP400000783	11:38717.6-38720.3	1950	1	649	71.20	5.09
StHsp70-14	PGSC0003DMP400052405	9:53571.5-53573.5	1722	0	573	62.41	5.53
StHsp70-15	PGSC0003DMP400019802	7:1089.7-1092.7	1845	1	614	67.47	5.11
StHsp70-16	PGSC0003DMP400015292	6:5489.7-5492.1	1461	1	486	53.27	5.48
StHsp70-17	PGSC0003DMP400048253	4:7035-7037.9	1950	1	649	71.23	5.07
StHsp70-18	PGSC0003DMP400021684	8:56395.4-56399.8	2004	7	667	73.46	5.04
StHsp70-19	PGSC0003DMP400032316	3:45718.2-45722.3	2007	7	668	73.61	5.10
StHsp70-20	PGSC0003DMP400042780	1:77390.8-77394.2	2010	6	669	74.68	5.32

ORF: MW: Molecular weight, pI: isoelectric points.

### Phylogenetic relationship, gene structure, and motif analysis

**Figure 1:**
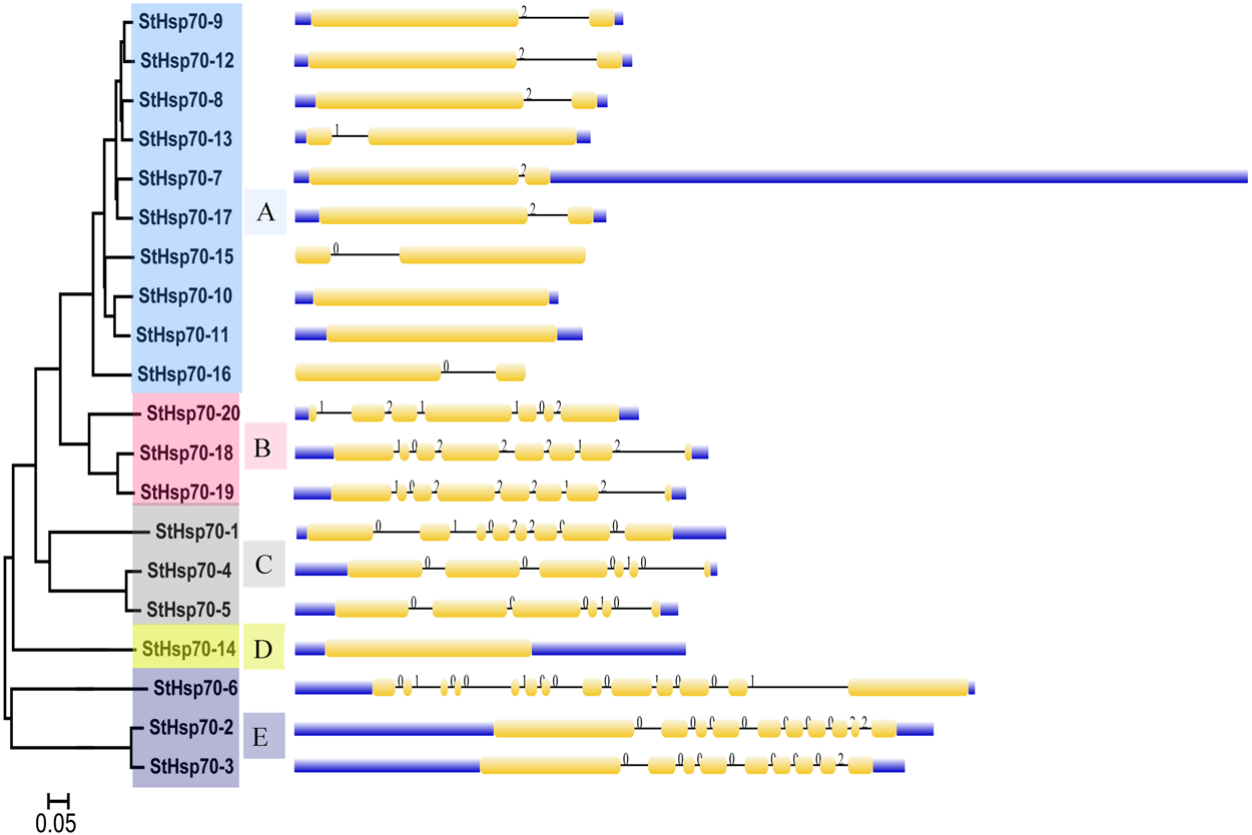
Phylogenetic relationships and gene structure of the potato *StHsp70* genes: The unrooted tree was generated using a MEGA5.0 program with the full-length amino acid sequences of the potato *StHsp70* proteins obtained by a Neighbor-Joining (NJ) method, including 1,000 boot-strap replications. The sub-families of *StHsp70* genes (**A**–**E**) are highlighted with different colored backgrounds. The exon/intron organization of the *StHsp70* genes is also shown as follows: The yellow boxes represent the exons; and the black lines represent the introns. The untranslated regions (UTRs) are indicated by the blue boxes. The numbers 0, 1, and 2 represent the splicing phases. The sizes of the exons and introns can be estimated using the scale detailed at the bottom.

In this research study, based on the phylogenetic tree of all the *StHsp70* protein sequences, the *StHsp70* gene family was divided into five subfamilies: Sub-families A, B, C, D, and E, respectively. Sub-family A had 10 members, and was the largest group. The Sub-families B, C, and E each contained three members, and Sub-family D had only one member (*StHsp70-14*) (Fig. 1). Additionally, Fig. 1 also provides a detailed illustration of the relative lengths of the introns, along with the conservation of the corresponding exon sequences within each *StHsp70* gene in the potato. The number of the introns was found to vary in the *StHsp70s*. All of the members in Sub-family A contained one intron, with the exception that no intron was found in two StHsp70 genes (*StHsp70-10* and *StHsp70-11*). Sub-family D only contained one member (*StHsp70-14*) without an intron. For Sub-families B and C, the number of introns ranged from 5 to 7, and Sub-family E contained more than 7 introns.

**Figure 2:**
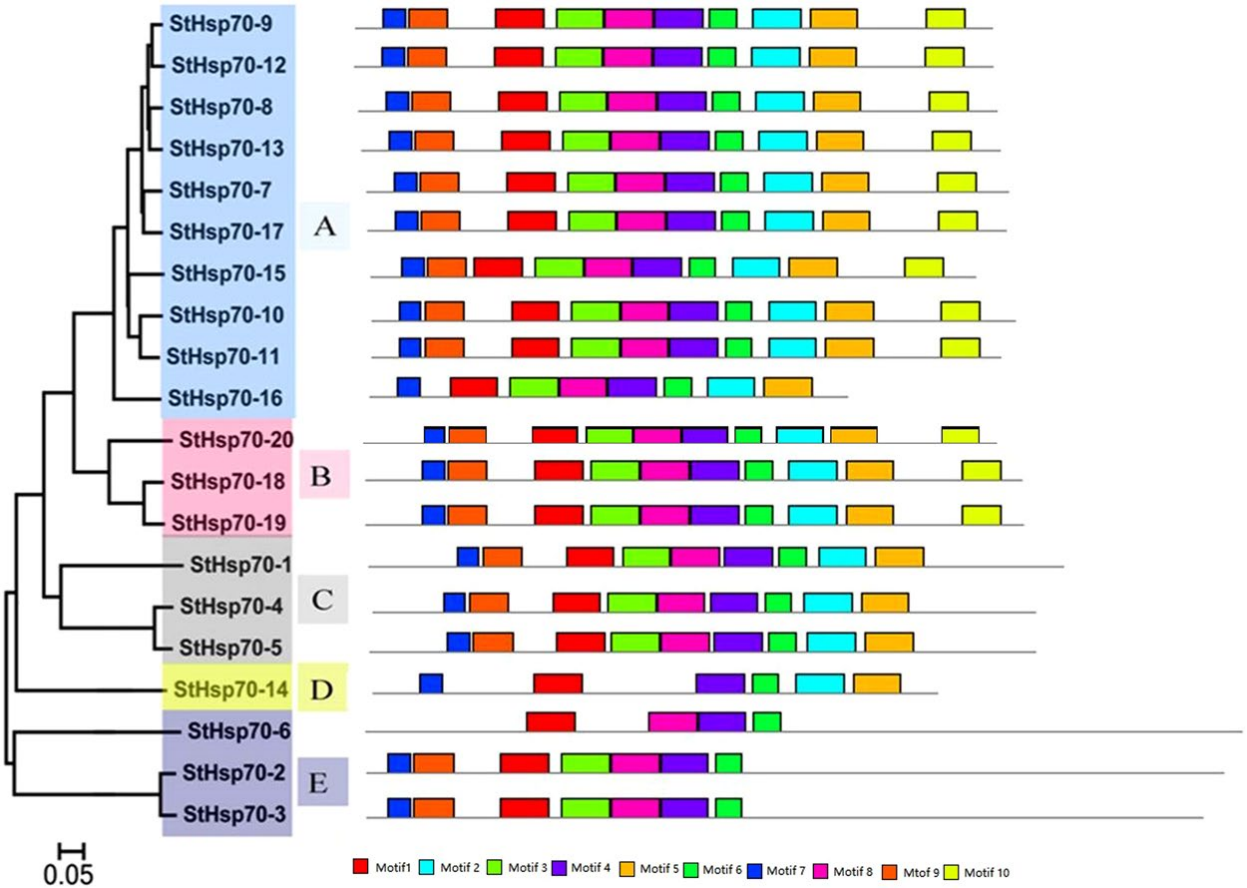
Conserved motifs in the *StHsp70* proteins: Each colored box represents a motif in each of the *StHsp70* proteins, with the motif’s name indicated in the box on the right.

In this study, the MEME of the total 20 *StHsp70* genes were also analyzed (Table 2, Fig. 2). The results showed the conserved domains or motifs were shared among related proteins, and 10 motifs in the *StHsp70* genes. Three motifs (Motif 1, Motif 3 and Motif 4) were found in all of the *StHsp70* genes. The motif 2 and Motif 5 were found in Sub-families A, B, C, and D. The Motif 10 was occurred in Sub-families A and B. In addition, two motifs (Motif 7 and Motif 8) were loss in *StHsp70-6* and *StHsp70-14*, respectively. Each of the motifs (Motif 3 and Motif 9) lacked both *StHsp70-6* and *StHsp70-14*. The type, order, and number of motifs were observed to be similar in the proteins within the same sub-family. However, they differed from the proteins in the other sub-families.

**Table 2:** Analysis of conserved motifs of *StHsp70* genes in potato.

Motif	Width	Best possible match
1	50	VKNAVVTVPAYFNDSQRQATKDAGVIAGLNVMRIINEPTAAAJAYGLDKK
2	50	PLSLGJETAGGVMTVLIPRNTTIPTKKEQVFSTYSDNQPGVLIQVYEGER
3	50	FDLGGGTFDVSJLTIEEGIFEVKATAGDTHLGGEDFDNRLVNHFVQEFKR
4	50	TRARFEELNMDLFRKCMEPVEKCLRDAKLDKSDIHEVVLVGGSTRIPKVQ
5	50	LGKFELSGIPPAPRGVPQIEVCFDIDANGILNVSAEDKTTGQKNKITITN
6	29	FNGKELCKSINPDEAVAYGAAVQAAILSG
7	24	VEIIANDQGNRTTPSYVAFTDTER
8	50	KHKKDISGBPRALRRLRTACERAKRTLSSTAQTTIEIDSLYEGIDFYSTI
9	41	GDAAKNQVAMNPENTVFDAKRLIGRRFSDPSVQSDMKLWPF
10	41	AVDZAIEWLDSNQLAEADEFEDKMKELESICNPIIAKMYQG

### Chromosomal locations of the *StHsp70* genes in potato

**Figure 3:**
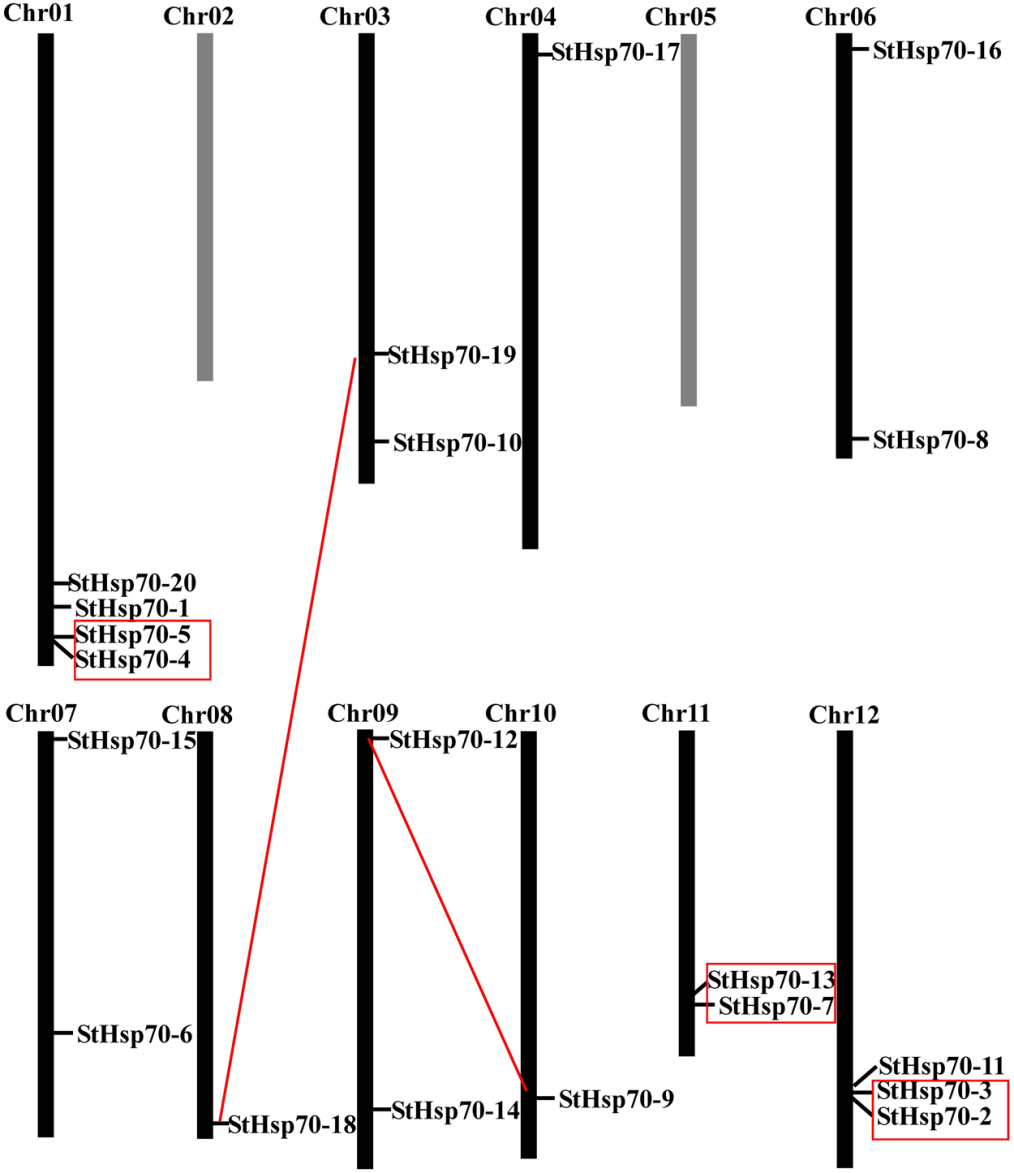
Chromosomal mapping of the *StHsp70* gene candidates in the potato. Segmentally duplicated genes are connected by red lines; Tandem duplicated genes are denoted by red boxes.

All of the *StHsp70* genes were found to be unevenly distributed on 10 of the 12 chromosomes in the potato genome. None of the *StHsp70* genes were mapped on chromosomes 2 and 5. Among the *StHsp70* genes, four genes were located in chromosome 1, and three *StHsp70* genes were positioned in chromosome 12. Also, chromosomes 4 and 10 each had only one *StHsp70* gene, and the remaining six chromosomes (3, 6, 7, 8, 9, and 11) each had two putative *StHsp70* genes (Fig. 3).

Additionally, the gene duplication events of the *StHsp70* family were also investigated in this study. The results showed that there were five sister pairs in the 20 *StHsp70* genes. Three of the five *StHsp70* paralog gene pairs were detected within a distance of less than 5 kb (>100 kb) on chromosomes 1, 11, and 12, respectively, which were identified as tandem duplications. The other two paralog gene pairs were determined to be segmental duplications. These results suggested that tandem and segmental duplications played a crucial role in the expansion of the HSP70 gene family in potato.

### Comparative analysis of the *StHsp70* genes in potato, tomato, Arabidopsis, and rice

**Figure 4:**
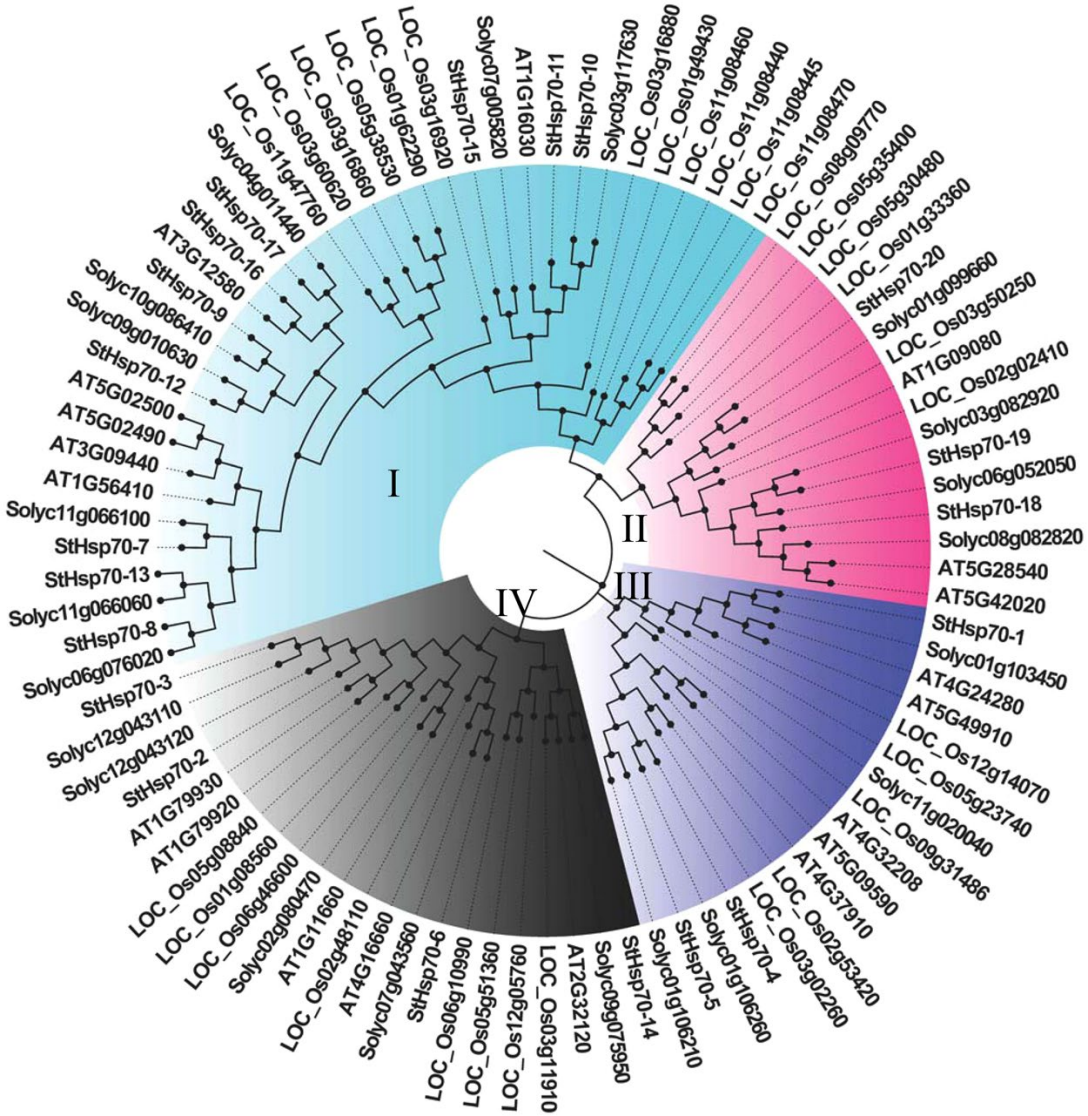
Phylogenetic tree of the *StHsp70* proteins from potato, tomato, Arabidopsis, and rice: All of the protein sequences were aligned using ClustalX1.83, and the phylogenetic tree was constructed in using a Neighbor-Joining (NJ) method with MEGA5.0 software. The boot-strap value was 1,000 replications.

In recent years, the development of comparative genomics has enabled the analyses of the same protein families among different plant species. In the current study, a phylogenetic tree was constructed which utilized 91 protein sequences from four different plant species, including potato (20), Arabidopsis (19), rice (31), and tomato (21), as detailed in Fig. 4. These were downloaded from the Heat Shock Protein Database Information Resource (http://pdslab.biochem.iisc.ernet.in/hspir/index.php). The *Hsp70* genes were separated into four groups: Groups I, II, III, and IV, respectively. Among these four groups, Group I was the largest with 36 members, which were composed of 10 members from potato, 12 from rice, eight from tomato, and six from Arabidopsis. Group II contained sixteen members, including three potato, six rice, four tomato, and three Arabidopsis members. Group III was composed by three potato, five Arabidopsis, five rice, and four tomato members. Class IV contained 22 members, including four potato, eight rice, five tomato, and five Arabidopsis members. All of the four sub-families contained rice, Arabidopsis, tomato, and potato *HSP70* genes, which suggested that the main characteristics of this family of plants had been generated prior to the dicot/monocot split.

The phylogenetic tree of the *HSP70* genes from the aforementioned four plant species also revealed their paralogous and orthologous relationships. The duplicated genes were contained in the paralogous pairs of each species, which supported the occurrence of species-specific *HSP70* gene duplication events. In this study, a total of twenty pairs of orthologous genes were identified, 16 of which were between potato and tomato; one pair was Arabidopsis (AT3G12580) with potato (*StHsp70-16*); another pair was from Arabidopsis (AT1G11660) and tomato (Solyc02g080470); and the last one was from Arabidopsis (AT2G32120) and rice (LOC_Os03g11910). Additionally, 12 pairs of parlogous genes were identified in this unrooted phylogenetic tree, which were determined to be from *Arabidopsis* and rice.

### Expression patterns of the *StHsp70* genes in different tissue

**Figure 5:**
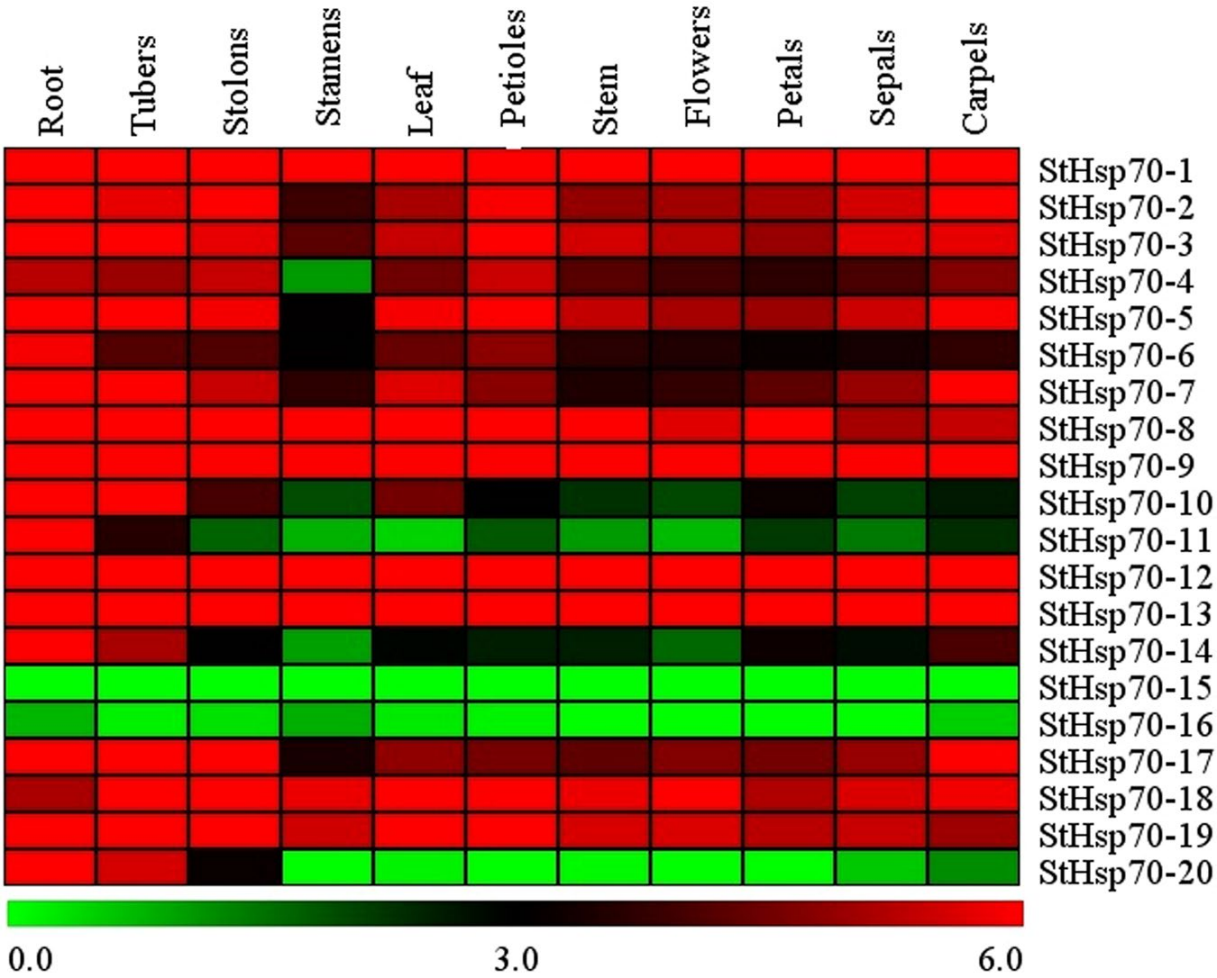
Expression profiles of the *StHsp70* genes in different tissue, including the roots, stems, and leaves, based on the RNA-seq (http://solanaceae.plantbiology.msu.edu/).

In this research study, in order to obtain the temporal and spatial expression profile of the *StHsp70* genes in different tissue, RNA-seq data were used to investigate the expression levels of these genes in the roots, leaves, tubers, stolon, stamens, petioles, flowers, petals, sepals, carpels, and stems (Fig. 5). The results showed that more than half of the *StHsp70* genes were highly expressed in all of the tested tissue. Furthermore, two genes *(StHsp70-15* and *StHsp70-16*) were lowly expressed or not expressed in these tissues. The expressions of the *StHsp70-11* and *StHsp70-20* genes were observed in two (root and tuber) and three (root, tuber, and stolon) of the tested tissue, respectively. The *StHsp70-4* gene was expressed in all of the tested tissue, with the exception of the stamen. The remaining two genes (*StHsp70-10* and *StHsp70-14*) were found to be highly expressed in the roots, tubers, stolon, and leaves, and lowly expressed in the stamen, stems, flowers, and sepals.

### qRT-PCR analysis of the *StHsp70* genes under abiotic stress conditions

**Figure 6:**
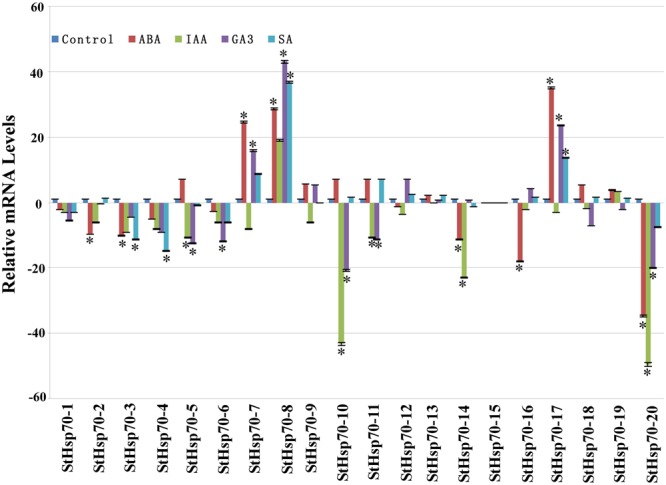
Expression of the potato *StHsp70* genes under abiotic stresses compared with untreated controls. The expression levels of these *StHSP70* genes in response to salt, drought, heat, and cold were tested using qRT-PCR. Expression levels were normalized using sesame β-actin as a reference gene^40^. Error bars represent standard deviations from three independent technical replicates. The mean expression levels from three independent biological replicates were analyzed for significance using t-tests (p < 0.05).

In this research study, for the purpose of gaining more insight into the roles of the *StHsp70* genes under abiotic stress conditions, the expression profiles of 20 *StHsp70* genes in response to drought, heat, cold, and NaCl stress conditions were analysed using qRT-PCR, and different expression profiles were observed (Fig. 6). The results showed that the expression levels of five of the *StHsp70* genes (*StHsp70-1*, *StHsp70-8*, *StHsp70-9*, *StHsp70-10*, and *StHsp70-17*) were up-regulated in four of the abiotic stress tests, and four genes were down-regulated (*StHsp70-2*, *StHsp70-4*, *StHsp70-13*, and *StHsp70-18*). Also, six genes (*StHsp70-3*, *StHsp70-5*, *StHsp70-6*, *StHsp70-12*, *StHsp70-14*, and *StHsp70-15*) were not found to be induced by these four abiotic stresses. The *StHsp70-7* gene was observed to be up-regulated under the drought stress conditions. The *StHsp70-11* gene was up-regulated by two abiotic stresses (salt and drought), while the *StHsp70-16* was up-regulated by the drought and cold stress conditions. The *StHsp70-20* genes exhibited high expression levels in three abiotic stresses (drought, heat, and cold), with the exception of the *StHsp70-19*, which was observed to be down-regulated by the heat and cold stress treatments.

### *StHsp70* expression under the condition of hormone treatments

**Figure 7:**
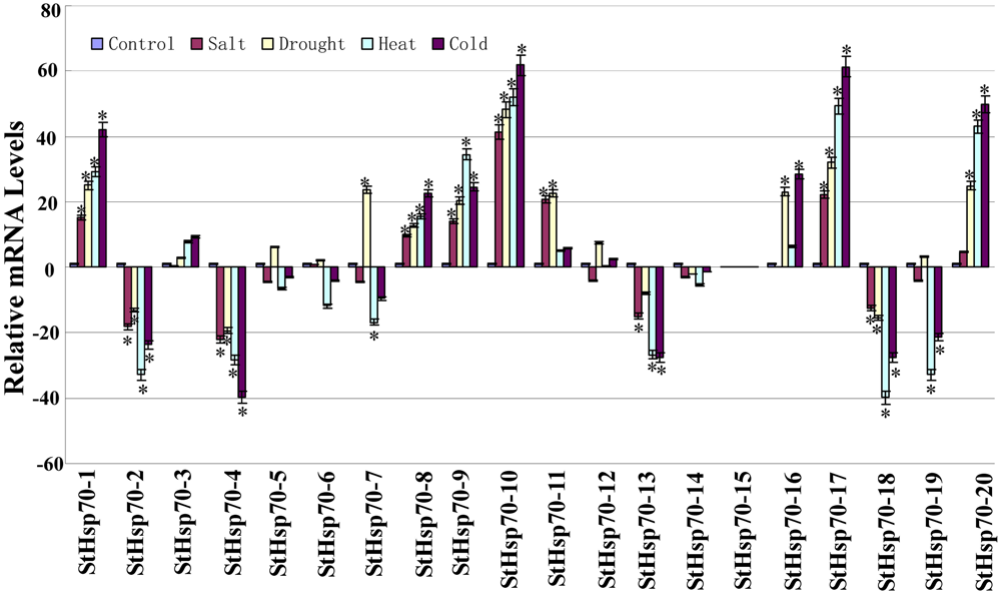
Expression profiles of the potato *StHsp70* genes under hormone treatments compared with untreated controls. The expression levels of these *StHSP70* genes in response to hormone stresses (IAA, ABA, GA3, and SA) were tested using qRT-PCR. Expression levels were normalized using sesame β-actin as a reference gene^40^. Error bars represent standard deviations from three independent technical replicates. The mean expression levels from three independent biological replicates were analyzed for significance using t-tests (p < 0.05).

Phytohormones IAA, SA, GA_3_, and ABA are known to be involved in the growth and development of plants. Therefore, in order to gain more insight into the molecular regulation of the *StHsp70* genes in response to hormone treatments, qRT-PCR was used to investigate the expression profiles of these genes under IAA, ABA, GA3, and SA treatments (Fig. 7). It was observed that, of all these *StHsp70* genes, the expressions of the 6, 8, 8 and 4 genes were changed (induced or repressed) under the IAA, ABA, GA3, and SA treatments, respectively. The number of down-regulated *StHsp70s* was significantly higher than that of the up-regulated genes under these hormone treatments. Furthermore, only one gene (*StHsp70-8*) was found to be highly expressed under the hormone treatment tests. Also, high expression levels of the genes (*StHsp70-7*) were observed under the ABA and GA3 treatments, and *StHsp70-17* was induced by all three hormones (ABA, GA3, and SA).

### Expression profiles of the *StHsp70* genes under *P*. *infestans* and the induced resistance chemical components

**Figure 8:**
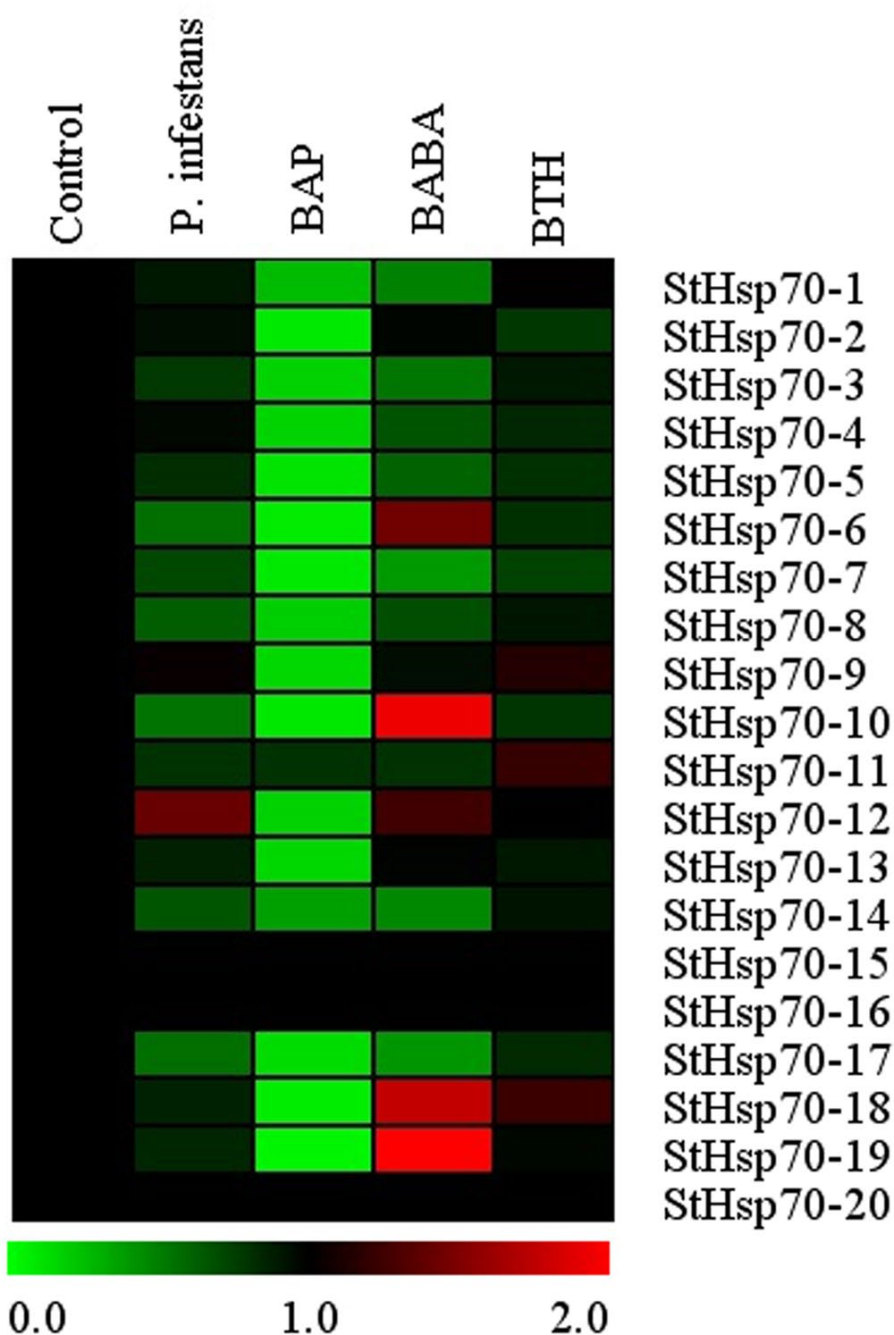
Expression profiles of the potato *StHsp70* genes in response to hormone stresses, including *P*. *infestans*, BAP, BABA, and BTH.

When invoked by the invasion of *P*. *infestans* and induced resistance chemical components, the transcript levels of all the *StHsp70* genes remained unaltered in the up- and down-regulated levels, respectively, of all of the tested samples (Fig. 8). In the *P*. *infestans* treatments, one of the *StHsp70* genes (*StHsp70-12*) was induced. For BABA treatments, nine of the twenty *StHsp70* genes were down-regulated, and five were up-regulated. In the BAP treatments, all of the *StHsp70* genes showed down-regulated levels, with the exception that the expressions of three *StHsp70* genes (*StHsp70-15*, *StHsp70-16*, and *StHsp70-20*) were observed to be unaltered. Under the BTH treatment conditions, all of the *StHsp70* genes were either down-regulated or displayed unaltered expression levels.

## Discussion

The *HSP70* gene family has previously been reported in several plants, including Arabidopsis, rice, soybean, and pepper^22–26^. However, this family had not previously been studied in potato. Therefore, this study performed a genome-wide analysis of the *HSP70* gene family in potato using a combination of the phylogeny, chromosomal locations, gene structures, conserved motifs, and expression profiles. Then, based on the phylogenetic tree and gene structures of the *StHsp70* proteins in potato, it was found that within the same sub-families, the most closely related *StHsp70* members shared similar exon/intron structures and intron numbers. These findings were determined to be consistent with the characteristics defined in the phylogenetic analysis (Fig. 1). For instance, in Sub-family A, all of the *StHsp70* members contained one intron, while those in Sub-family B contained 6 to 7 introns. In Sub-family E, the number of intron ranged from 8 to 12. These findings indicated that some intron loss and gain events may have occurred during the structural evolution among the potato *StHsp70* genes. In the motif analysis, the same situation was also revealed. The type, order, and number of motifs in the *StHsp70* proteins within the same sub-family were observed to be similar, but differed from the proteins within the other sub-families.

It is known that the duplications of individual genes, chromosomal segments, and entire genomes have been major forces in the evolution of plant genome structures^27^. In this study, in order to understand the expansion mechanism of the potato *StHsp70* gene family, the gene duplication events (tandem and segmental duplications) were investigated. Previous research studies have reported that a tandem duplication event can be confirmed by the presence of two or more genes on the same chromosome, while a segmental duplication event is defined as gene duplications on different chromosomes^28^. In the potato genome, two pairs of *StHsp70* members (*StHsp70-18*/*StHsp70-19*; and *StHsp70-9*/*StHsp70-12*) were identified as segmental duplication genes. Meanwhile, three pairs of *StHsp70* members (*StHsp70-4*/*StHsp70-5*; *StHsp70-7*/*StHsp70-13;* and *StHsp70-2*/*StHsp70-3*) were identified as tandem duplication genes. Therefore, it was indicated that segmental and tandem duplication events play important roles in the expansion of the *StHsp70* gene family.

Previous studies have also reported that *HSP70* genes play important roles in the responses of plants to various stress conditions, such as drought, salt, and heat^29^. In this study, the expression profiles of the *StHsp70* genes under salt, drought, heat, and cold stresses (Fig. 6) were examined. The results revealed that the majority of the *StHsp70* genes in potato were induced by these four abiotic stresses. In particular, almost half of the *StHsp70* genes showed the same expression profile, and were either significantly up-regulated (5) or down-regulated (4) under all of the tested abiotic stress conditions. These results indicated that the genes may potentially play shared roles in abiotic stress resistance. However, it was observed that the expression profiles of the genes responding to the different stress conditions usually tended to be different. For instance, drought-regulated genes (for example, *StHsp70-7*) were down-regulated under salt, heat, and cold stress conditions, which indicated different signing pathways. All of these results also implied that the signaling pathways which were mediated by the abiotic stresses in the plants were very complicated systems. In addition, this study further showed that 13 of the 20 *StHsp70* genes were induced by *P*. *infestans*, which indicated that they could participate in mediating the disease resistance in plants in response to biotic stresses. In this study, several of the *StHsp70* genes were also found to be induced by chemical components (BAP, BABA, and BTH). All of the results suggested that these genes could play potential roles in enhancing broad-spectrum resistance to late blight conditions. In this research study, the identification of the *StHsp70s* with potential value for the improvement of stress resistance in the potato will benefit from targeting the genes which are known to be part of the abiotic response networks.

We also found that most *StHSP70s* are regulated by both abiotic stresses (salt, drought, heat and cold) and hormone treatments (ABA, IAA, GA3 and SA), and exhibit a similar trend (Figs 6 and 7). This supported the view that some these environment stresses share similar regulating responses and signal transduction pathways^30^. In addition, previous researchers reported that ABA takes part in various stress responses including drought^31^. In our study, three StHSP70s were up-regulated by ABA and drought and demonstrated a similar trend. These findings suggested a common and positive role in the ABA and drought stress signaling pathways.

## Conclusions

In this research study, a total of 20 *StHsp70* genes were identified in potato using bioinformatics methods. The analysis of their genome distributions, gene organizations, and gene structures suggested a complex evolutionary history for this family in potato. In the potato genome, segment/tandem duplications were determined to have greatly contributed to expansion of *StHsp70* gene family. Also, based on RNA-seq data, the results revealed that they participated in the growth and development of the plants. The expression profiles of the *StHsp70* genes in response to abiotic stress conditions demonstrated that this gene family was widely involved in the salt, drought, heat, and cold stress responses. Furthermore, more than half of *StHsp70* genes were found to be induced by *P*. *infestans* and chemical components, which indicated an important role in the broad-spectrum resistance to late blight. The overall analysis of this gene family will potentially assist in facilitating further research regarding the evolutionary history and biological functions of the *Hsp70* gene family.

## Materials and Methods

### Genome-wide identification of the *HSP70* proteins in potato

In this study, the potato genome sequence was downloaded from the SPud DB Potato Genomics Resources (http://solanaceae.plantbiology.msu.edu/), and then used to construct a local database with BioEide 7.0 software. The HMM profile of the *HSP70* domain (Pfam: PF00012) from Pfam (http://www.sanger.ac.uk/Software/Pfam) was utilized to search the candidate potato *HSP70* proteins. The putative potato *HSP70* protein sequences were submitted to blast against the potato genome in the NCBI (http://blast.ncbi.nlm.nih.gov/blast.cgi), and SPud DB Potato Genomics Resources (http://solanaceae.plantbiology.msu.edu/). The E-value was sets as <10^−5^. All of the obtained protein sequences were examined for the presence of the *HSP70* domain using SMART (http://smart.embl-heidelberg.de/) tools^32^.

All of the potato *HSP70* genome information, including the chromosomal location data, protein sequences, and CDS sequence, were downloaded from the website SPud database (http://solanaceae.plantbiology.msu.edu/). The protein sequences were analyzed using EXPASY PROTOPARAM (http://www.expasy.org/tools/protparam.html) in order to obtain the number of theoretical amino acid, molecular weight, isoelectric point, and instability index considered value. A ProtParam Tool (http://web.expasy.org/portparam) was used to calculate the theoretical PI and Mw^33^.

### Phylogenetic analysis

The Arabidopsis, rice, tomato, and pepper *HSP70* protein sequences were downloaded from the Heat Shock Protein Database Information Resource (http://pdslab.biochem.iisc.ernet.in/hspir/index.php). The multiple sequence alignment of these *HSP70* protein sequences were performed using CLUSTALX 2.0 software with default parameters. The phylogenetic tree was constructed using MEGA 6.0 software with a Neighbor-Joining method, and a bootstrap analysis was conducted using 1,000 iterations with a pair-wise gap deletion mode^34^.

### Chromosomal locations and gene duplications

The chromosomal location images of the potato *HSP70* genes were generated by MapChart software, in accordance with the chromosomal position information provided in the potato gene database (http://solanaceae.plantbiology.msu.edu/). Then, in order to identify whether tandem and segmental duplication events had occurred, two genes in the same species, which were located in the same clade of the phylogenetic tree, were defined as being coparalogs^35^. Then, the SPud browser (http://solanaceae.plantbiology.msu.edu/ was used to detect the coordinates of the segmental duplications of the target genes. The coparalogs were deemed be the results of the segmental duplications if they were located on duplicated chromosomal blocks^35^. Additionally, the paralogs were determined to be tandem duplicated genes if two genes were separated by five or fewer genes in a 100 kb region^36^. The local alignments of two protein sequences were calculated using the Smith-Waterman algorithm (http://www.ebi.ac.uk/Tools/psa/).

### Gene structures and motif analyses

The analyses of the intron/exon organization of the potato *HSP70*, based on their genomic and CDS sequences, were performed using the online tools of the Genes Structure Display Server program (GSDS, http://gsds.cbi.pku.edu.cn/index.php)^37^. The conserved motifs of the potato *HSP70* were determined using a Multiple EM for motif elicitation (MEME) (http://meme.nbcr.net/meme3/meme.html)^38^. The parameters were as follows: Number of repetitions: any; maximum number of motifs: 10; and optimum motifs: 6 to 200 amino acid residues.

### Gene expression analysis based on the RNA-seq data

In this research study, in order to gain the family-level expression patterns of the *StHsp70* genes, RNA-based data analyses of both the development and biotic stress responses were performed. Then, the transcriptome data of the genome-wide gene expression atlas of potato were downloaded (http://solanaceae.plantbiology.msu.edu/pgsc_download.shtml) for the purpose of analyzing the spatial and temporal expression profiles of the *StHsp70* genes during development. The gene expression data from *P*. *infestans* infections, as well as the data from induced resistance chemical component treatments, were also downloaded (http://solanaceae.plantbiology.msu.edu/pgsc_download.shtml). To render the data suitable for cluster displays, absolute FPKM values were divided by the mean of all of the values, and the ratios were transformed by log2. Then, the expression data were hierarchically clustered based on the Euclidean distance, with an average linkage in MeV 4.5^39^.

### Growth conditions and treatments of the potato

In this study, a 168 R potato breeding line was selected. In the spring of 2016, seeds of the 168 R line were sterilized for five minutes using a 10% hypochlorous acid solution, and washed three times with distilled water. The seeds were then placed on water-saturated filter paper to germinate, and cultivated in a Hoagland solution in a growth chamber which was maintained under the conditions of 16 hours of light at 25 °C, and 8 hours of dark at 18 °C. The relative humidity was maintained between 60 to 80%. For the salt and drought stress treatments, seedlings at the fourth true leaf were transferred to 300 mM NaCl or 400 mM mannitol for four hours. For the cold and heat treatments, the seedlings were kept at 4 ± 1 °C and 42 ± 1 °C, respectively, for two hours. The seedlings which were kept in water for the same duration at 25 ± 1 °C served as the control. For the hormone treatments, the seedlings at the second true leaf stage were sprayed with solutions of naphthylacetic acid (IAA, 10 μM), salicylic acid (SA, 100 μM), methyl jasmonic acid (MeJA, 100 μM), or absciscic acid (ABA, 100 μM), and then sampled two hours later. For the induced resistance chemical component treatment, the seedlings were sprayed with BAP (10%), BABA (10%), and BTH (10%) in sterile water. The control plants were sprayed with sterile water only. All of the samples were frozen in liquid N_2_, and stored at −80 °C. Also, three biological replicates were performed in this study’s experiment.

### qRT-PCR analysis

The RNA was isolated from all of the samples (leaves, roots, and stems) using Trizol reagent (Invitrogen, USA), which was followed by a DNaseI (Promega, USA) treatment, for the purpose of removing any traces of genomic DNA. The first-strand cDNA synthesis was performed using a Transcript First-strand cDNA synthesis SuperMix Kit (Transgen, Beijing). The gene-specific primers were designed using Primer 5.0 (Supplemental Table S1). The relative expressions of each of the selected genes were normalized using the reference gene *EF1α*^40^. Then, real-time PCR was conducted utilizing an Applied Biosystems StepOne Real-Time PCR System (Applied Biosystem, USA). The volume consisted of a 10 μL SYBR premix Taq (2×) mixture, and 1 μL of cDNA. The upstream and downstream primers were both 0.4 μL and 8.2 μL of ddH_2_O, for a total volume of 20, with a FastStart Universal SYBR Green Master, followed a cycling profile as follows: 95 °C for 10 minutes; 40 cycles at 95 °C for 15 seconds, and 60 °C for 60 seconds (all of the reactions were run in triplicate). The expression levels were calculated as the mean signal intensity across the three replicates. Relative gene expression was calculated using ΔΔCT values obtained from the formulas ΔCT = CT target-CT reference and ΔΔCT = ΔCT treated sample-ΔCT untreated sample (0-h treatment). For all chart preparations, the calculation of the gene expression levels followed the 2^−△△CT^ method described by Livak and Schmittgen^41^. Also, three technical replicates were carried out for each of the samples.

### Statistical analysis

In this study, all the data were calculated using Student’s t-test at a significance level of 0.05 in Microsoft Excel software. All of the expression analyses were performed for three biological replicates, and the values shown in the figures represent the average values ±standard deviation (SD) of three replicates. Differences between values (P < 0.05) test was used to identify significant differences among the treatments.

## Electronic supplementary material

Supplementary Table S1
