# Supplementary material for: The Hsp70 Gene Family in Solanum tuberosum: Genome-Wide Identification, Phylogeny, and Expression Patterns
Source: Sci Rep. 2018 Nov 9;8:16628. doi: 10.1038/s41598-018-34878-7 (PMC6226454; doi:10.1038/s41598-018-34878-7)
Supplement: Supplementary file 1 — Supplementary Table S1 [file 41598_2018_34878_MOESM1_ESM.doc]

**The *Hsp70* Gene Family in *Solanum tuberosum*: Genome-Wide Identification, Phylogeny, and Expression Patterns**

Jia Liu^1,2^, Xin Pang^3^, Yuan Cheng^4^, Yuhe Yin^1^, Qiang Zhang^1^, Wenbin Su^2^, Bing Hu^1^, Qinwei Guo^5^, Si Ha^2^, Jianping Zhang^2^*, Hongjian Wan^4^*

^1^Wulanchabu Academy of Agricultural and Husbandry Sciences, Wulanchabu, 012000, Inner Mongolia, China

^2^Plant Protection Institute, Inner Mongolia Academy of Agricultural and Animal Husbandry Sciences, Hohhot, Inner Mongolia, 010031, China

^3^Suzhou polytechnic institute of agriculture, Suzhou, 215008, Jiangsu, China

^4^State Key Laboratory Breeding Base for Zhejiang Sustainable Pest and Disease Control, Institute of Vegetables, Zhejiang Academy of Agricultural Sciences, Hangzhou, China

^5^Quzhou Academy of Agricultural Sciences, Quzhou, 324000, Zhejiang, China

f

Supplementary Table S1 Specific primer for qRT-PCR of *StHSP70* genes.

| Gene | Forward primer | Reverse primer |
| --- | --- | --- |
| StHSP70-1 | CACATTGCCTGGTGATGAGG | CTCTTTCACTGGCCCTGGTA |
| StHSP70-2 | AAGTACGCCTAACCCTGCAT | CCATTCTGGCAGGTCCCTTA |
| StHSP70-3 | AGAAGGCAGAGGCACTTGAT | AGGAGATTGAGGTGGTGGTG |
| StHSP70-4 | AGAAGGCAGAGGCACTTGAT | AGGAGATTGAGGTGGTGGTG |
| StHSP70-5 | GTGCTAAATGGGCTGGTCTG | GCAACACAAGAGTTGGTGGT |
| StHSP70-6 | TGAAGGATGCTGTGGTGACT | TGTTCATTCACAAGCGCCAA |
| StHSP70-7 | CTGCTTCGACATTGATGCCA | GGATAGTCGACCCTTGTCGT |
| StHSP70-8 | AGGGATGCCAAGATGGACAA | AACAGCCTCATCTGGGTTGA |
| StHSP70-9 | CCGTCCTACGTTGGTTTCAC | CCAGGACCAGGAATGACCTT |
| StHSP70-10 | ATGGCATTCTCAACGTGTCG | ACCTCTCAGCCTCTTGAACC |
| StHSP70-11 | TCGGTCGACGTTTCTCTGAT | TCTCCTCTGGTGCAAACTGT |
| StHSP70-12 | CTCTGCAAGAGCATCAACCC | TCATGACACCTCCAGCAGTT |
| StHSP70-13 | AGGGATGCCAAGATGGACAA | AACAGCCTCATCTGGGTTGA |
| StHSP70-14 | ATGGGATTGCTTCGAGTTGC | TCTTCAAACTCTGCCCTCGT |
| StHSP70-15 | ACTTGAAACTGCTGGTGGTG | GTGCTGGAGGGATACCAGAG |
| StHSP70-16 | GAGGGAGAGAGAGCAAGGAC | CCTCTGTCCGGTGGTATTGT |
| StHSP70-17 | ACCTCTTTCCCTTGGTCTGG | ACCAGGTTGATTGTCGGAGT |
| StHSP70-18 | CCTTGGTGGTGGGACATTTG | CTCACCTCCAAGATGGGTGT |
| StHSP70-19 | CTGGTTTGAACGTGGCAAGA | ACCACCACCAAGGTCAAAGA |
| StHSP70-20 | GATGCACAAAGGCAAGCAAC | CAAATGTGCCACCACCAAGA |
